# Familial Mediterranean Fever and Human autoinflammatory diseases

**DOI:** 10.1186/1546-0096-13-S1-P71

**Published:** 2015-09-28

**Authors:** L Hovhannisyan

**Affiliations:** 1 Yerevan State Medical University, Yerevan, Armenia

## Introduction

Human Autoinflammatory Diseases (HADs) is a heterogeneous group of rare genetic diseases, which are characterized by unprovoked onsets of inflammation, fever and clinical symptoms analogous with rheumatic diseases with absence of immunological indicators. Familial Mediterranean Diseases (FMF) is one of the popular forms in the group of syndromes which are called HPFS.

Clinical characterization is presented in symptoms which are common for all hereditary fevers like: relapsing onsets of inflammation (serous membrane) of muscular-joint syndrome, various skin rash, high rates of inflammatory processes (ESR, leucocytosis, c-reactive protein, SAA), secondary amyloidosis complications, absence of autoantibodies. Absence of specific symptoms of manifestation hampersdifferential diagnosis. The used diagnostic criteria are insufficient for timely diagnosis of Familial Mediterranean Diseases, especially in case of atypical debut of disease.

It is actualforthe group of Hereditary Periodic Fever Syndromes and Human Autoinflammatory Diseases, such as Still's disease, Crohn's disease, Bekhchet's disease and Juvenile Idiopathic Arthritis (JIA) that are characterized by the multifactor types of inheritance.

## Aim

In spite of the existing data onthe pathogenic relationship between FMF and the above mentioned diseases, it is still unclear whether they are concomitant with FMR or are associated with FMR.

## Methods

Via retrospective analysis we examined medical history of 24 patients for a period of 2011-2013 (medical histories taken from the archive of “Muratsan” clinical hospital).

## Results

Below isthe description of two cases of patients diagnosed with FMF, which started with Crohn's disease and JIA.

Case 1. Patient A., 22 years old. The disease started up in childhood with symptoms of arthritis, fever, hepatosplenomegaly and skin rash. The patient was diagnosed with JIA and steroid treatment was prescribed. At the age of 14 attack-like pain appeared in the abdomen and chest, accompanied with fever. Periodicity of symptoms was twice a month. Based on the clinical picture (serositis, synovitis, fever) and genetic investigation (M694V), the FMR was diagnosed in 2012. The colchicines therapy was prescribed.

Case 2. Patient S., 60 years old. The disease started upin childhood with acute symptoms of abdominal pain which was not always accompanied with fever. 15 years later symptoms of Crohn's disease developed, such as diarrhea, intestinal bleeding, joint syndrome (sacroileitis and talocrural arthritis). The patient was hospitalized with the nephritic syndrome. Based on the clinical picture and genetic investigation (M694V in homozygote condition) and biopsy of rectum (amyloidal deposits were discovered), the abdominal form of FMR, systemic amyloidosis with affection of kidneys and intestine were diagnosed.

## Conclusion

Summarizing the above mentioned, we have come to the conclusion that there is need to do genetic investigation of MEFV in the population ethnically significant for FMR, when inflammatory intestinal diseases and clinical pictures of JIA are registered.

## Consent to publish

Written informed consent for publication of their clinical details was obtained from the patient/parent/guardian/relative of the patient.

